# Ischemic stroke patients are biologically older than their chronological age

**DOI:** 10.18632/aging.101028

**Published:** 2016-08-25

**Authors:** Carolina Soriano-Tárraga, Eva Giralt-Steinhauer, Marina Mola-Caminal, Rosa M. Vivanco-Hidalgo, Angel Ois, Ana Rodríguez-Campello, Elisa Cuadrado-Godia, Sergi Sayols-Baixeras, Roberto Elosua, Jaume Roquer, Jordi Jiménez-Conde

**Affiliations:** ^1^ Department of Neurology, Hospital del Mar; Neurovascular Research Group, IMIM (Institut Hospital del Mar d'Investigacions Mèdiques); Universitat Autònoma de Barcelona/DCEXS-Universitat Pompeu Fabra, Barcelona, Spain; ^2^ Cardiovascular Epidemiology and Genetics Research Group, IMIM, Barcelona Spain; ^3^ Universitat Pompeu Fabra, Barcelona, Spain

**Keywords:** Ischemic stroke, aging, epigenetics, biological age, DNA methylation

## Abstract

Ischemic stroke is associated with aging. It is possible to predict chronological age by measuring age-related changes in DNA methylation from multiple CpG sites across the genome, known as biological age. The difference between biological age and actual chronological age would indicate an individual's level of aging. Our aim was to determine the biological age of ischemic stroke patients and compare their aging with controls of the same chronological age. A total of 123 individuals, 41 controls and 82 patients with ischemic stroke were paired by chronological age, ranging from 39 to 82 years. Illumina HumanMethylation450 BeadChip array was used to measure DNA methylation in CpG sites in both groups, and biological age was estimated using methylation values of specific CpGs. Ischemic stroke patients were *biologically* an average 2.5 years older than healthy controls (p-value=0.010). Stratified by age tertiles, younger stroke patients (≤57 years old) were biologically older than controls (OR=1.19; 95%CI 1.00-1.41, p-value=0.046). The older groups showed no biological age differences between cases and controls, but were close to reaching the significance level. Ischemic stroke patients are *biologically* older than controls. Biological age should be considered as a potential new biomarker of stroke risk.

## INTRODUCTION

Ischemic stroke (IS) is a complex age-related disease with high mortality and long-term disability. Despite current attention to risk factors and preventive treatment, the number of stroke cases has risen in recent decades, likely because the aging population has increased. Stroke pathogenesis involves a number of different disease processes as well as interactions between environmental, vascular, systemic, genetic, and central nervous system factors [1].

In recent years, however, incidence of stroke has also increased among younger adults [2,3]. Approximately 10% of IS occurs in individuals younger than 50 years, which is called “young stroke” [4,5]. This increase is often attributed to a high prevalence of unusual, rare conditions or to nontraditional risk factors such as migraine, illicit drug use, oral contraceptives, pregnancy and patent foramen oval. In older patients, stroke remains associated with the traditional risk factors: hypertension, hypercholesterolemia, diabetes mellitus, and obesity [6].

The epigenetic marker that has been studied most extensively is DNA methylation (DNAm), which is essential for regulation of gene expression. This mechanism consists of the covalent addition of a methyl group to a cytosine nucleotide, primarily in the context of a CpG dinucleotide. This dinucleotide is quite rare in mammalian genomes (∼1%) and is clustered in regions known as CpG islands. Methylation of the CpG island is associated with gene silencing. DNAm is dynamic, varies throughout the life course, and its levels are influenced by lifestyle and environmental factors, as well as by genetic variation [7]. Given its dynamic nature, epigenetics has been referred to as the interface between the genome and the environment [8].

Age-related changes in DNA methylation are well documented, and two recent studies used methylation measured from multiple CpGs across the genome to predict chronological age in humans [9,10]. Hannum et al. [9] created an age predictor from whole blood DNA, based on a single cohort of 656 individuals aged 19 to 101 years. Horvath developed a multi-tissue age predictor using DNA methylation data from multiple studies [10]. Both models are based on the Illumina BeadChip. The difference between chronological age and methylation-predicted age, defined as average age acceleration (Δ_age_), can be used to determine whether the DNAm age is consistently higher or lower than expected. These age predictors are influenced by clinical and lifestyle parameters, they are predictive of all-cause mortality, indicating that they are more suggestive of biological age than of chronological age [11–14].

Age is one of the main risk factors for stroke. We hypothesized that biological age would be even more closely associated with stroke risk, and that “young stroke” patients may be undergoing accelerated aging, with a higher biological than chronological age.

## RESULTS

We examined a cohort of 123 individuals, 41 controls and 82 patients with IS, matched by chronological age.

The clinical and demographic characteristics of the study population are shown in Table 1.

**Table 1:** Descriptive characteristics of study participants. Summary details of DNAm age predicted with Hannum and Horvath methods and differences between chronological age and DNAm age.

	Controls	IS	p-value
	N=41	N=82	
Age*	62.8(14.2)	63.9 (10.3)	0.621
Sex, female, n (%)	20 (48.8)	37 (45.1)	0.701
Hyperlipidemia	20 (48.8)	44 (53.7)	0.610
Hypertension, n (%)	19 (46.3)	54 (65.9)	0.038
Diabetes, n (%)	7 (17.1)	30 (36.6)	0.026
Coronary heart disease, n (%)	1 (2.4)	8 (9.8)	0.142
Atrial fibrillation, n (%)	2 (4.9)	24 (29.3)	0.002
BMI, Kg/m^2^‡	28.3 (4.8)	29.0 (5.2)	0.523
Smoking habit, n (%):			
Current/Former (<5 years)	20 (48.8)	47 (57.3)	0.370
Never smokers	21 (51.2)	35 (42.7)	
Ischemic stroke etiology, n (%)			
Large-artery atherosclerosis	-	9 (11.0)	
Small-artery disease	-	39 (47.6)	
Cardioembolism	-	34 (41.5)	
Hannum DNAm age (years)	63.9 (11.9)	67.2 (8.8)	0.081
Hannum difference	1.1 (5.5)	3.3 (5.7)	0.041
Horvath DNAm age (years)	58.2 (10.8)	60.7 (10.0)	0.204
Horvath difference	−4.6 (5.8)	−3.2 (7.6)	0.300

* Mean (Standard deviation)

† Median (Interquartile range)

‡ BMI, Body Mass Index.

We initially used two approaches described in the literature to predict biological age, the Hannum and Horvath methods. The average biological age of controls showed a mean Hannum-predicted age higher than their chronological ages by a mean of 1.1 years (SD=5.5); their Horvath-predicted age was lower than their chrono-logical ages by 4.6 years (SD=5.8). In patients with IS, we observed a Hannum-predicted age higher than their chronological age by a mean of 3.3 years (SD=5.7), statistically significant compared to controls (p-value=0.04). Their Horvath-predicted age was lower than their chronological ages by 3.2 years (SD=7.6) (Table 1).

**Figure 1:**
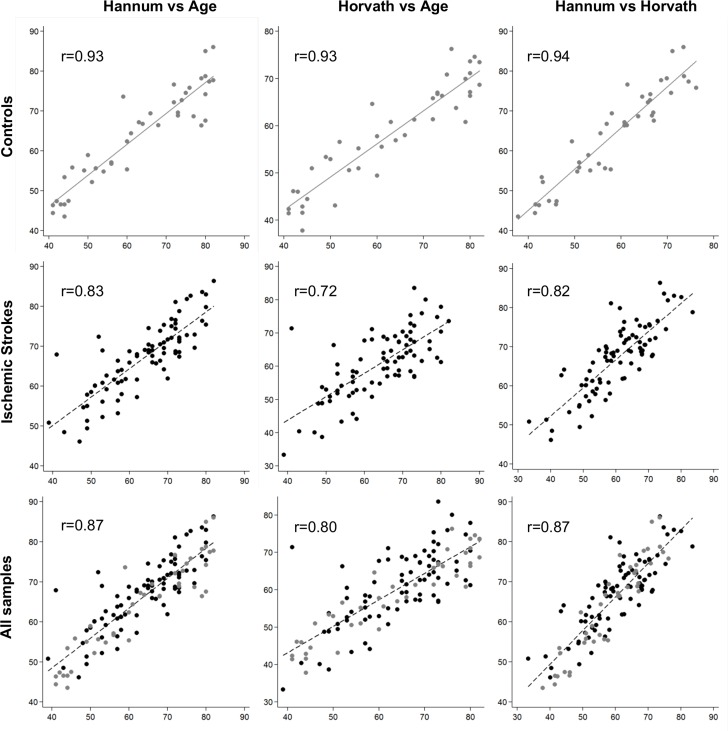
Plots of predicted methylation age (Hannum and Horvath) against chronological age and plots of Hannum versus Horvath predicted methylation age. r, Pearson correlation.

DNAm age had a strong positive correlation with chronological age in control samples (0.93 for both Hannum and Horvath methods, and 0.94 between the Hannum- and Horvath-predicted ages). In IS cases, the correlations were lower (0.83 for the Hannum method, 0.72 for the Horvath method, and 0.82 between the two (Figure 1). Although both age predictors showed high accuracy in our samples, Hannum DNAm age performed better, with fewer differences in chrono-logical age in controls and better correlation in patients with IS than the Horvath method.

**Figure 2:**
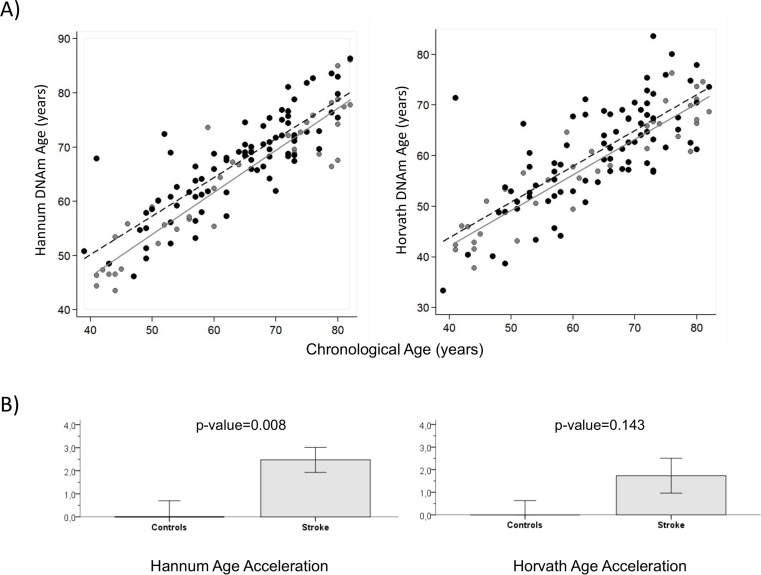
DNA methylation Age (**A**) Hannum and Horvath versus chronological age in blood samples. Grey and black circles in the scatterplot denote samples from controls and cases, respectively. The grey line represents a linear regression line through control samples. The black dashed line represents a linear regression line through IS cases. For each subject, age acceleration is defined as the vertical distance to the grey line. The bottom row (**B**) shows how mean age acceleration (y-axis) relates to IS status, with p-value of ANOVA test. By definition, the mean age in controls is zero. Each bar plot reports 1 SE.

Regression lines through sample values stratified by IS cases and controls suggest that IS cases have a higher DNAm (biological) age than controls using both the Hannum and Horvath methods (Figure 2-A). Moreover, we calculated average age acceleration (Δ_age_) for both age predictor methods, defined by the average difference between DNAm age and chronological age. Δ_age_ is not correlated with chronological age (Δ_age_ Hannum, r=-0.07, p=0.47; Δ_age_ Horvath, r=0.009, p=0.92), on the other hand it is correlated to stroke status with Hannum Δ_age_ (r=0.24, p=0.008) and close to significance with Horvath Δ_age_ (r=0.133 p=0.143). ANOVA test showed that biological age of IS cases was older than controls, with a significant average Hannum Δ_age_ of 2.5 years (SD=4.9), p=0.008 and Horvath Δ_age_ of 1.7 years (SD=7.0), p=0.143, close to significance (Figure 2-B).

Four logistic regression models were considered. Model 1 was the raw model with no adjustments. Model 2 was logistic regression model adjusted by sex, because sex variable was significantly associated to Δ_age_ Hannum (p-value=0.029). Finally, model 3 (full model) was adjusted by sex, smoking habit, atrial fibrillation, hypertension and diabetes mellitus. According to these models, IS cases showed a significant Hannum Δ_age_ effect, being biologically older than controls of the same chronological age (Table 2). The adjusted model 3 showed OR=1.14 (95%CI 1.03-1.26), p-value=0.015. Model 4 adjusted by model 3 variables and blood cell proportion associated Hannum and Horvath DNAm age (Supplemental Table S1). Hannum Δ_age_ was statistically significant OR=1.13 (95%CI 1.003-1.26), p-value=0.045. The Horvath method yielded results close to statistical significance in all four models, a trend similar to the Hannum results.

**Table 2:** Logistic models that regress IS status on age acceleration (model 1), adjusted by sex (model 2), adjusted by sex, diabetes mellitus, hypertension, atrial fibrillation, and smoking habit (model 3) and adjusted by sex, diabetes mellitus, hypertension, atrial fibrillation, smoking habit and blood cell count associated to Hannum (NK, monocytes, CD4+ T cells, naïve CD8 T cells and CD8+CD28-CD45RA-) and Horvath (NK, monocytes, CD8+ T cells, naïve CD8 and CD4 T) (model 4). OR: Odd Ratio; CI: confidence interval.

	Model 1		Model 2		Model 3		Model 4	
Control vs IS	OR (95%CI)	p-value	OR (95%CI)	p-value	OR (95%CI)	p-value	OR (95%CI)	p-value
**Δ_age_Hannum**	1.13 (1.03-1.23)	0.010	1.13 (1.03-1.24)	0.010	1.13 (1.02-1.24)	0.015	1.13 (1.003-1.26)	0.045
**Δ_age_ Horvath**	1.05 (0.98-1.12)	0.145	1.05 (0.98-1.12)	0.151	1.07 (0.99-1.15)	0.086	1.06 (0.97-1.15)	0.223

**Figure 3:**
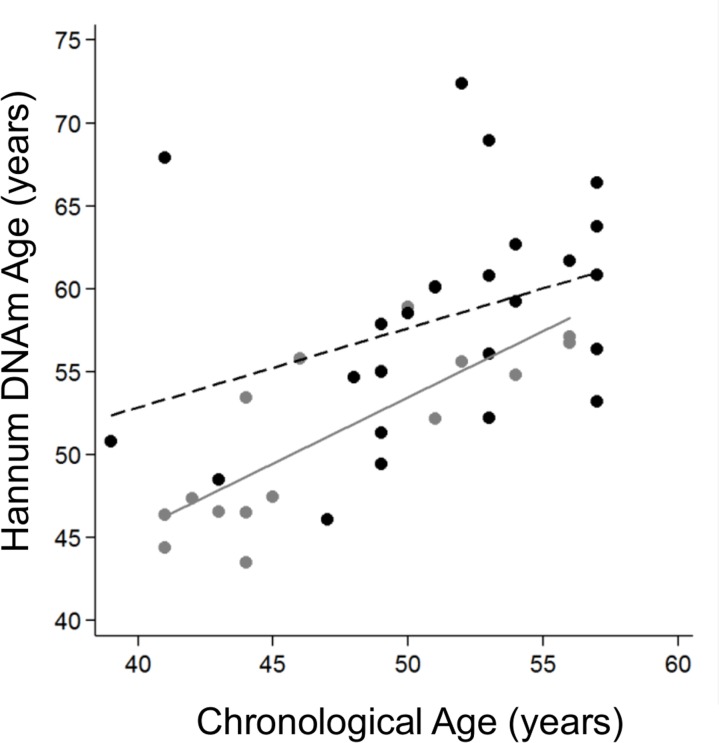
Hannum DNA methylation Age versus chronological age of younger adults (≤57 years old). Grey and black circles in the scatterplot denote samples from controls and cases, respectively. The grey line represents a linear regression line through control samples. The black dashed line represents a linear regression line through IS cases, OR=1.13 (95%CI 1.03-1.23), p-value 0.01.

We stratified the cohort by age tertiles, considering *young* individuals those ≤57 years old, *mid-aged* between 58-71 years old and *elderly* as ≥72 years old, and grouped the *mid-aged* and *elderly* groups together as *older adults* to get more statistical power. The clinical and demographic characteristics by age group are shown in Table 3. We continued our study using the Hannum age predictor because it was statistically significant in the previous analysis. Logistic analysis revealed that the biological age of young IS cases was significantly older than controls for both model 1 (OR=1.19 [95%CI 1.00-1.41], p-value=0.041) and model 2, (OR=1.19 [95%CI 1.00-1.41], p-value=0.046) (Figure 3), but not for the fully adjusted model (OR=1.13 [95%CI 0.97-1.32], p-value=0.131). The older IS groups were more similar to the control group in biological age, but the difference was close to reaching significance (Table 4).

**Table 3:** Descriptive characteristics of the participants by age tertiles, comparing ≤57 versus ≥ years old (the other two tertiles).

	Young ≤57 years			Elderly ≥58 years		
	Controls	IS	p-value	Controls	IS	p-value
N	16	25		25	57	
Sex, female, n(%)	7 (43.8)	5 (20)	0.103	13 (52.0)	32 (56.1)	0.729
Hyperlipidemia, n(%)	6 (37.5)	14 (56)	0.248	14 (56.0)	30 (52.6)	0.814
Hypertension, n(%)	3 (18.8)	14 (56)	0.018	16 (64.0)	40 (70.2)	0.580
Diabetes, n(%)	1 (6.3)	6 (24)	0.141	6 (24.0)	24 (42.1)	0.117
Coronary heart disease, n(%)*	0	1 (4.0)	0.418	1 (4.0)	7 (12.3)	0.245
Atrial fibrillation, n (%)	0	3 (12)	0.150	2 (8.0)	21 (36.8)	0.007
BMI, Kg/m^2^†	26.7 (22.3-30.8)	26.6 (24.0-30.9)	0.808	29.4 (26.5-31.8)	28.3 (25.6-33.0)	0.690
Smoking habit, n (%):						
Current/Former (<5 years)	11 (68.8)	22 (88)	0.129	9 (36)	25 (43.9)	0.506
Never	5 (31.3)	3 (12)		19 (64)	32 (56.1)	

* Mean (Standard deviation)

† Median (Interquartile range)

‡ BMI, Body Mass Index.

**Table 4:** Logistic models that regressed IS status, stratified by age, on Hannum age acceleration (model 1), adjusted by sex (model 2), and adjusted by sex, diabetes mellitus, hypertension, and smoking habit (model 3). Atrial fibrillation is not included in the model because control cohort data did not show cases. OR: Odd Ratio; CI: confidence interval

IS status By Age	Model 1		Model 2		Model 3	
	OR (95%CI)	p-value	OR (95%CI)	p-value	OR (95%CI)	p-value
All	1.13 (1.03-1.23)	0.010	1.13 (1.03-1.24)	0.010	1.13 (1.02-1.24)	0.015
≤57	1.19 (1.01-1.41)	0.041	1.19 (1.00-1.41)	0.046	1.13 (0.97-1.32)	0.131
≥58	1.09 (0.98-1.22)	0.111	1.11 (0.99-1.24)	0.083	1.12 (0.99-1.27)	0.064

## DISCUSSION

The sensitivity analysis evaluating which age predictor performed better in our study determined that the Hannum predictor was superior. This is likely because this method is constructed on the basis of DNA methylation data from whole blood, like our data, while the Horvath method is constructed on a range of different tissues and cell types [11].

A growing body of evidence suggests that many manifestations of aging are epigenetic [15,16]. In particular, DNA methylation associates with chronological age over long time scales and changes in methylation have been linked to complex age-associated diseases such as metabolic disease and cancer [17–23].

One possible explanation for this association could lie in the accumulation of environmental exposures that may contribute to epigenetic changes with age. Changes in the functionality of epigenetic machinery in addition to exposure of the genome to environmental factors could contribute to increasing epigenetic diversity with age [24,25]. Many studies have found a mean decrease in blood DNA methylation with increasing age. It has been reported that DNA obtained from a 103-year-old donor was more unmethylated overall than DNA from the same cell type obtained from a neonate, showing that DNA methylomes differ at the two extremes of the human lifespan [26,27]. Environmental and stochastic factors are associated to epigenetic changes with age, and two events, epigenetic drift and epigenetic clock, contribute to age-related DNA methylation changes.

Epigenetic drift represents the tendency for increasing discordance between epigenomes over time but these differences are not consistent across individuals [27]. In fact, has been found that methylation marks in identical twins differ increasingly as a function of age, both in genome variation and lifetime environmental exposures [18,28]. On the other hand, the epigenetic clock represents those sites that are associated with age across individuals and can be used to predict the age of an individual [24,29–31].

Incidence of IS has been strongly correlated with increasing age [32]. Approximately 10% to 15% of all IS occurs in young adults. The prevalence of standard modifiable cardiovascular risk factors in young stroke patients differs from that in older patients, with the prevalence of hypertension, diabetes mellitus, obesity, lipid disorders, congenital heart disease, and smoking increasing with age [2,32,33]. In this context, the increase in biological age, compared to healthy controls, echoes the burden of exposures —vascular risk factors, lifestyle habits, environment-- that leads to an epigenetic profile similar to elderly people.

Previous studies have shown that epigenetic age relates to cognitive status, physical fitness, mortality, HIV infection, Parkinson Disease and Down Syndrome [11,34–38]. Our group previously reported a global hypomethylation in IS cases compared to controls and age-associated DNA hypomethylation using the LUMA assay [39]. Now, we find for the first time that epigenetic age in patients with IS averages 2.5 years older than in controls, but is especially patenting in young stroke patients.

A strength of our study is that we evaluated two distinct epigenetic biomarkers of aging. The association between age acceleration and IS was stronger for the blood-based Hannum method, although the multitissue-based Horvath method showed similar (but nonsignificant) results. Moreover, this is the first study to determine the biological age of stroke patients.

Some limitations of the study should be considered. Although the sample size was appropriate to detect findings consistently and significantly, the study may be underpowered to reach statistical significance for some analyses with the Horvath age predictor or further stratified sub-analyses. Second, we measured DNA methylation in peripheral blood cells. Methylation levels of some CpGs/regions are tissue-specific [40] and we might have lost some signals by not choosing specific tissues where biological aging could have a higher impact on risk for stroke. However, methylation patterns of whole blood have been reported as a good proxy for methylation levels from a specific site of action [41,42].

In conclusion, we found that IS status was associated with a significant increase in Hannum DNA methyla-tion, likely as a consequence of the accumulation of cardiovascular risk factors, and near signification with Horvath method. Patients with IS were biologically older than controls, a difference that was more obvious in young stroke. This could open up the possibility of useful new biomarker of stroke risk.

## METHODS

### Study participants

The study included Caucasian patients prospectively recruited and analyzed retrospectively.

From 2009 to 2012, 82 patients with IS where recruited in Hospital del Mar in Barcelona, Spain, and were included in the BASICMAR Register (Ministerio de Sanidad y Consumo, Instituto de Salud Carlos III; FIS No. PI051737) [43,44]. Inclusion criteria in BASICMAR cohorts were as follows: (1) first-ever IS, (2) brain imaging with CT or MRI, (3) availability of clinical data supporting the assigned stroke subtype according to TOAST classification [45], (4) absence of intracranial hemorrhage, neoplasms, demyelinating and autoimmune diseases, and vasculitides. All patients were assessed and classified by a neurologist and were included in the study by consecutive order of recruitment.

Control samples (N=41) were obtained from the Girona Heart Registry (REGICOR, which stands for REgistre GIroni del COR), a population-based cohort recruited in the province of Girona, about 100 km from Barcelona. This register includes a randomized representative sample of men and women of the province [46].

The study was approved by the local ethics committee, CEIC-Parc de Salut Mar, and participants gave written informed consent. The study was conducted according to the principles expressed in the Declaration of Helsinki and relevant legislation in Spain.

### Demographic and vascular risk factor variables

In accordance with international guidelines, data on vascular risk factors analyzed were obtained from direct interview of the patient, relatives and caregivers, and from medical records. Examinations were performed and standardized questionnaires administered during the hospitalization by a team of neurologists and reviewed by an additional neurologist.

We recorded age, sex, and vascular risk factors using a structured questionnaire, as follows: arterial hypertension (HT), defined as systolic blood pressure (SBP) ≥140 mmHg or diastolic (DPB) ≥90 mmHg recorded from more than 2 measurements previous to the acute event, a physician's diagnosis, or use of medication; hyperlipidemia, defined as a physician's diagnosis, use of medication, serum cholesterol concentration >220 mg/dL, low-density lipoprotein cholesterol (LDL) >130 mg/dL, or serum triglyceride concentration >150 mg/dL; diabetes mellitus (DM), defined as evidence of two or more fasting blood glucose values≥126 mg/dl, use of diabetes medication, or a physician's diagnosis; coronary heart disease (CHD), defined as documented history of angina pectoris or myocardial infarction; atrial fibrillation (AF) (documented history or diagnosis during hospitalize-tion); and self-reported smoking habit. During hospitalization, lymphocyte count and body mass index (BMI) were recorded and TOAST criteria were used to classify IS subtype [45], according to standardized protocol.

### Peripheral blood collection and DNA extraction

DNA samples were extracted from whole peripheral blood collected in 10 mL EDTA tubes. The Chemagic Magnetic Separation Module I system (Chemagen) and the Autopure LS (Qiagen) were used for DNA isolation in the BASICMAR patient cohort; only the Autopure LS (Qiagen) was used in the REGICOR cohort. In both cohorts, genome-wide DNA methylation was assessed using the Illumina HumanMethylation450 Beadchip. DNA extractions were performed at the same time and stored together at -20°C. DNA concentrations were quantified using the Picogreen assay and Nanodrop technology. The quality of DNA samples was visualized in agarose gels. We tested whether DNA isolation method was a confounder in our methylation analysis; no statistical differences were observed.

### Array-based DNA methylation analysis with Infinium HumanMethylation 450k

Genomic DNA (1 μg) was bisulfite-converted using the EZ-96 DNA Methylation Kit (Zymo Research, Orange, CA, USA) according to the manufacturer's procedure and recommended alternative incubation conditions when using the Illumina Methylation Assay.

Genome-wide DNA methylation was assessed using the Illumina HumanMethylation450 Beadchip (Illumina Netherlands, Eindhoven, Netherlands) following the manufacturer's protocol with no modifications. The arrays were scanned with the Illumina HiScan SQ scanner. These processes were carried out in Progenika Biopharma in Bizkaia, Spain.

### Data pre-processing and normalization

Data were pre-processed using standardized pipelines [47,48]. Sample and CpG quality controls and the statistical analysis were performed as described in Soriano-Tarraga et al [44]. Initial quality control of sample data was conducted using GenomeStudio version 2011.1 (Illumina, San Diego, CA, USA). Before analysis methylation values were corrected for background values and then normalized by *SWAN* using *minfi* Bioconductor package [48] and then, β values were transformed using a variance stabilization transformation to methylation M-values.

We used a previously published Houseman algorithm to infer white blood cell counts from DNA methylation data [51] and Horvath online calculator [10].

### DNA methylation age and epigenetic clock

DNA methylation (DNAm) age, also known as “epigenetic age” or “biological age”, was calculated using the DNA methylation levels of whole blood DNA. Two measures of DNA methylation age were calculated. The Horvath method uses 353 probes common to the Illumina 27 K and 450 K Methylation arrays using data from a range of tissues and cell types [10]. Methylation age was determined using the online calculator (https://labs.genetics.ucla.edu/horvath/htdocsdnamage/). The Hannum method is based on 71 methylation probes from Illumina 450 K Methylation array, derived as the best predictors of age using data generated from whole blood [9]. DNA methylation age was calculated as the sum of the beta values multiplied by the reported effect sizes for the Hannum predictor.

The concept of age acceleration (Δ_age_) is defined as the residuals from the linear regression of DNAm age on chronological age in control samples [11]. This measure of Δ_age_ is not correlated with chronological age and takes on a positive value for samples whose DNAm age is higher than expected.

### Statistical analysis

Baseline characteristics of IS patients and controls were compared using t-test for continuous variables and chi-square for categorical variables. We calculated the Pearson correlation of DNAm age and chronological age for all the samples and stratified by IS patients and controls. Sensitivity analysis was carried out to assess which age predictor performed better in our study, in order to simplify and improve the statistical model.

Four logistic models were analyzed. In Model 1 (raw model), IS status was regressed on Hannum age acceleration. Model 2 was model 1 adjusted by sex. Model 3 was model 1 adjusted by atrial fibrillation, diabetes mellitus, hypertension, and smoking habit, and stratified by age using tertile cut points (≤57,58-71 and ≥72 years). Moreover, model 4 was performed, adjusted by previous variables (atrial fibrillation, diabetes mellitus, hypertension, and smoking habit) and blood cell proportion associated to Hannum age acceleration (NK, monocytes, CD4+ T cells, naïve CD8 T cells and CD8+CD28-CD45RA) and Horvath age acceleration (NK, monocytes, CD8+ T cells, naïve CD8 and CD4 T cells). We tested for differences in DNAm Age using all four logistic models

All statistical analyses were performed using R statistical package, version 3.2 [52], STATA and SPSS version 18.0. The following packages were utilized in R: *minfi, sva* and *limma* [49,53,54]. Statistical significance was set at a p-value of 0.05.

## SUPPLEMENTARY MATERIAL TABLE


