# Supplementary material for: Ischemic stroke patients are biologically older than their chronological age
Source: Aging (Albany NY). 2016 Aug 25;8(11):2655–65. doi: 10.18632/aging.101028 (PMC5191861; doi:10.18632/aging.101028)
Supplement: Supplementary file 1 [file aging-08-2655-s001.pdf]

SUPPLEMENTARY MATERIAL

Table S1. Correlation of chronological age, Hannum and Horvath mAge with blood cell types.

	Controls (n=41)			IS (n=82)		
	Age	mAge Hannum	mAge Horvath	Age	mAge Hannum	mAge Horvath
CD4T	-0.36 p=0.02	-0.29 p=0.07	0.02 p=0.90	-0.30 p=0.006	-0.38 p<0.001	-0.12 p=0.27
CD8T	-0.15 p=0.34	-0.08 p=0.62	0.27 p=0.09	-0.02 p=0.87	0.02 p=0.87	0.28 p=0.01
NK	0.50 p=0.01	0.04 p=0.80	0.26 p=0.10	0.16 P=0.14	0.05 p=0.64	0.21 p=0.05
Bcell	-0.34 p=0.03	0.09 p=0.57	0.14 p=0.38	0.01 P= 0.90	-0.02 p=0.87	-0.12 p=0.29
Monocytes	0.17 p=0.30	0.23 p=0.14	-0.09 p=0.56	0.05 p=0.66	0.12 p=0.29	0.20 p=0.07
Granulocytes	0.12 p=0.45	0.22 p=0.17	-0.31 p=0.053	0.04 p=0.69	0.15 p=0.19	-0.12 p=0.28
Naïve CD8	-0.62 p<0.001	-0.15 p=0.35	-0.22 p=0.18	-0.35 p=0.001	-0.40 p<0.001	-0.22 p=0.05
Naïve CD4	-0.52 p=0.001	-0.23 p=0.16	0.05 p=0.74	-0.37 p=0.001	-0.38 p=0.011	0.04 p=0.73
PlasmaBlast	0.24 p=0.125	0.13 p=0.41	-0.16 p=0.33	0.07 p=0.55	0.11 p=0.34	-0.13 p=0.25
CD8+CD28-CD45RA-	0.38 p=0.02	-0.16 p=0.33	0.18 p=0.27	0.35 p=0.001	0.23 p=0.037	0.15 p=0.18
